# Primary hyperparathyroidism in a 4-month-old German Shepherd mixed breed dog

**DOI:** 10.1093/jvimsj/aalag247

**Published:** 2026-10-10

**Authors:** Juan Aires-Serrano, Jerry Shimali, Jade Renard, Frances Bird, Alenka Hrovat

**Affiliations:** Pride Veterinary Referrals, Surgery Department, Derby DE24 8HJ, United Kingdom; Oakwood Veterinary Referrals, Internal Medicine Department, Hartford CW8 1LP, United Kingdom; Vet Oracle Teleradiology, Norfolk IP22 4ER, United Kingdom; Pride Veterinary Referrals, Surgery Department, Derby DE24 8HJ, United Kingdom; Oakwood Veterinary Referrals, Internal Medicine Department, Hartford CW8 1LP, United Kingdom

**Keywords:** canine, hypercalcemia, parathyroid, pediatric

## Abstract

A 4-month-old female-intact German Shepherd mixed breed dog was evaluated for polyuria, polydipsia, oral discomfort, tachypnea, and pyrexia. Blood testing documented total and ionized hypercalcemia with an increased parathyroid hormone concentration. Computed tomography identified bone abnormalities of the mandibule and mild, diffuse pulmonary changes. Ultrasonography of the neck documented one mildly enlarged parathyroid gland that was surgically removed. Histopathology indicated diffuse chief cell hyperplasia. The ionized calcium concentration normalized within 7 days postoperatively.

## Introduction

Primary hyperparathyroidism (PHPT) is an endocrine condition predominantly diagnosed in older dogs.^1–3^ The disease commonly develops as a result of a solitary adenoma (80-85% of dogs) with autonomous secretion of parathyroid hormone (PTH). Parathyroid gland hyperplasia and malignant neoplasia are less common (5%‑6%).^1^^,^^2^^,^^4^^,^^5^ Most dogs show nonspecific signs, including lethargy, muscle weakness, polyuria, polydipsia, anorexia, weight loss, constipation, vomiting, and lower urinary tract signs.^1–4^ In dogs, the disease is diagnosed by documenting increased total and ionized calcium (iCa) concentrations, or rarely ionized hypercalcemia alone, and a serum PTH concentration that is increased or in the upper half of the reference range with concurrent hypercalcemia.^6^^,^^7^ Diagnosis requires the exclusion of other conditions causing hypercalcemia. After laboratory confirmation, enlarged parathyroid glands are identified by ultrasonography of the neck. Glands > 3 mm at their largest dimension are considered enlarged.^5^ Parathyroidectomy is the most effective treatment; ultrasound-guided radiofrequency heat or ethanol ablation are alternative treatments.^8–10^

Keeshounds are an overrepresented breed affected by PHPT, but the disease can occur in any breed.^3^^,^^11^ A genetic test for PHPT predisposition in Keeshounds has been developed,^12^ but performance data have not been published. Heritability has not been proven in other breeds.

Pediatric PHPT in humans is very rare, with skeletal and renal abnormalities predominating.^13^ Bone pain, an increased risk of fractures and skeletal deformities, nephrocalcinosis, and nephrolithiasis are common.^14–16^ Enlarged glands in children are identified by ultrasonography of the neck and scintigraphy, and the standard treatment is surgery, with the most common histologic diagnosis being a solitary parathyroid adenoma.^13^

Juvenile PHPT has been described as a hereditary condition in German Shepherd dogs (GSD). One study described PHPT in 2 GSD puppies with abnormal growth, lameness, muscular weakness, polydipsia, and polyuria. Both puppies had increased total calcium and PTH concentrations, and hereditability was suspected because both affected dogs came from the same litter.^17^

We report the clinical, laboratory, imaging, and histologic findings in a surgically treated 4-month-old GSD mixed breed with PHPT.

## Case history

A 4-month-old female-intact GSD mixed breed was referred for a 2-day history of lethargy, marked polyuria and polydipsia, pyrexia, discomfort on oral examination and vomiting. No relevant dietary history was reported. The dog was fed a combination of commercial dry and wet puppy food with no access to raw food. Blood test results from the referring practice identified increased total calcium concentration (3.58 mmol/L; reference interval [RI], 1.95‑3.15), mild hyperglobulinemia (41 g/L; RI, 23‑38), total proteins (73 g/L; RI, 48‑72), mild anemia (hematocrit 0.35 L/L; RI, 0.38‑0.56), a decreased mean corpuscular hemoglobin concentration (30.8 g/dL; RI, 32.6‑39.2), increased total white blood cell count (29.1 × 10^9^/L; RI, 4.9‑17.6), neutrophil count (24.74 × 10^9^/L; RI, 2.94‑12.67), and the presence of band neutrophils (0.58 × 10^9^/L; RI, 0.00‑0.17). The body condition score was 3/9 with mild pelvic limb and epaxial muscle atrophy. The dog appeared uncomfortable during the oral examination but could open its mouth fully. Thoracic auscultation and abdominal palpation were unremarkable. Bilateral mild postural carpal valgus was noted, but the range of motion was normal (Figure 1). No spinal or joint abnormalities or pain were noted.

**Figure 1:**
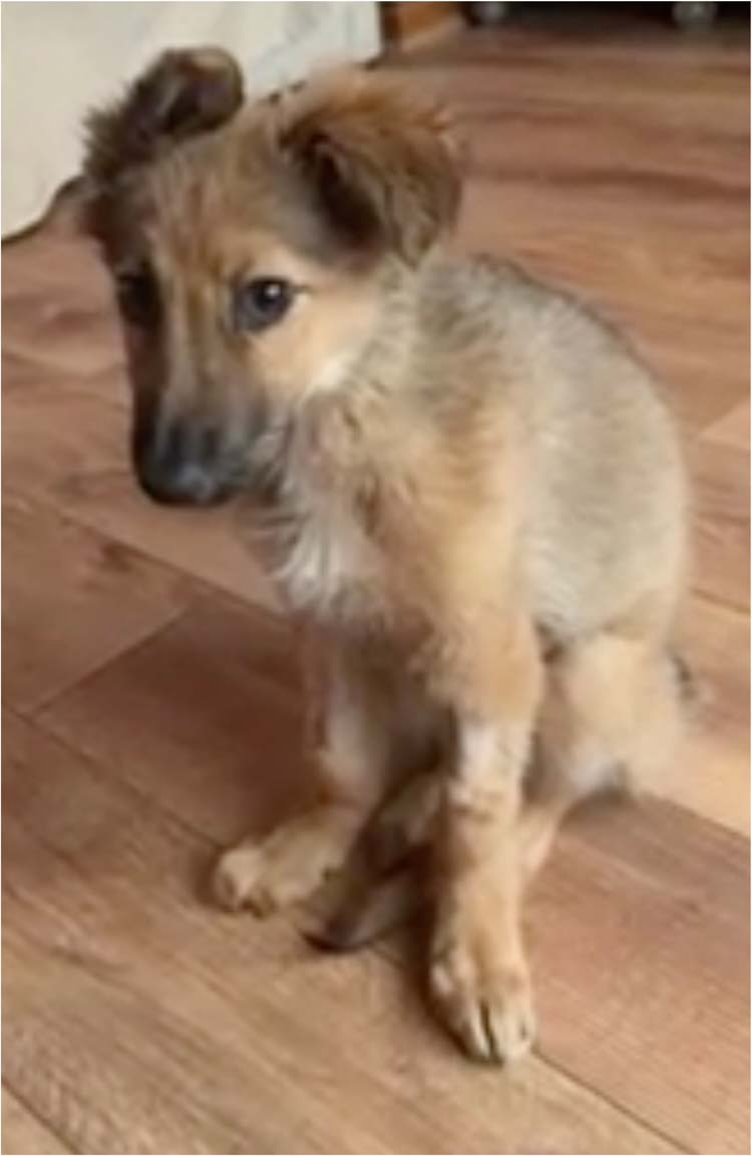
Patient in sitting position. Valgus deformity of the left carpus observed.

Venous iCa concentration at presentation measured using a blood gas analyzer (iSTAT Alinity Abbott Park, IL, USA) was increased (2.17 mmol/L; RI, 1.21‑1.45), and the serum sodium concentration was decreased (135 mmol/L; RI, 141‑150). The basal serum cortisol concentration was 320.05 nmol/L, and the C-reactive protein (CRP) concentration was increased (96.3 mg/L; abnormal > 30). Blood was submitted for PTH, PTH-related-protein (PTHrP) and 25-OH-vitamin D measurements. Results indicated an increased PTH concentration (78 pg/ml; RI 20‑65); PTHrP and 25-OH-vitamin D concentrations were both normal: < 0.1 pmol/l (RI normal < 0.5) and 92 nmol/L (RI 88—257), respectively.

The dog was premedicated with butorphanol (*Torbugesic,* Zoetis, Girona, Spain; 0.2 mg/kg IV) and medetomidine (*Sedator,* Dechra, Bladel, The Netherlands; 3 μg/kg IV) and induced with propofol (*Propofol**®*** Lipuro Vet Virbac, Melsungen, Germany; to effect IV). Anesthesia was maintained with isoflurane and oxygen. The oral examination was unremarkable. Urine was collected by cystocentesis and submitted for urinalysis and culture, identifying a urine specific gravity 1.010, 3+ hemoglobinuria, 5‑10 leukocytes per high power field (hpf), 50‑100 erythrocytes per hpf, 15‑20 epithelial cells per hpf, and 1+ bacteriuria. The urine protein:creatinine ratio was increased at 3.71 (normal, < 0.2), and the urine culture was negative.

Computed tomography (CT; GE Health Care Fixed Revolution® ACT) was performed using iohexol (*Omnipaque,* GE Healthcare, Oslo, Norway; 2 mL/kg IV) for contrast. Bilateral small subchondral bone defects were identified along the articular surface of the mandibular condyles, and an irregular bone surface with partial cortical discontinuity was noted along the mandibular neck and ramus (Figures 2 and 3). Diffuse ground-glass attenuation of the lung parenchyma and bronchial wall thickening were also noted. Computed tomography scan of the abdomen was unremarkable.

**Figure 2:**
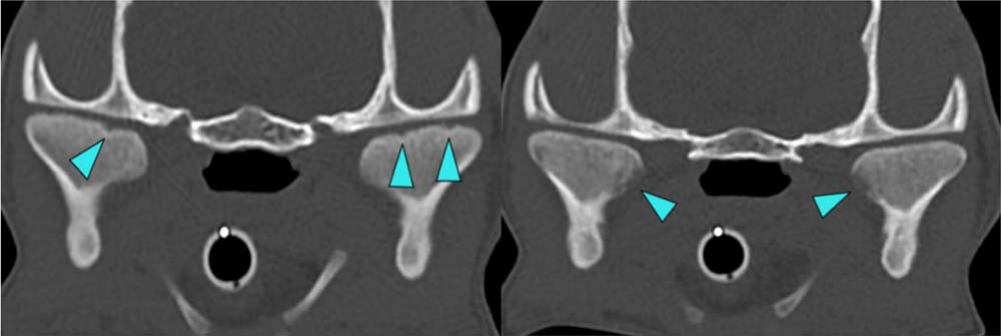
Sequential skull computed tomography scan images in the transverse plane. Arrows indicate small subchondral bone defects along the articular surface of the mandibular condyles.

**Figure 3:**
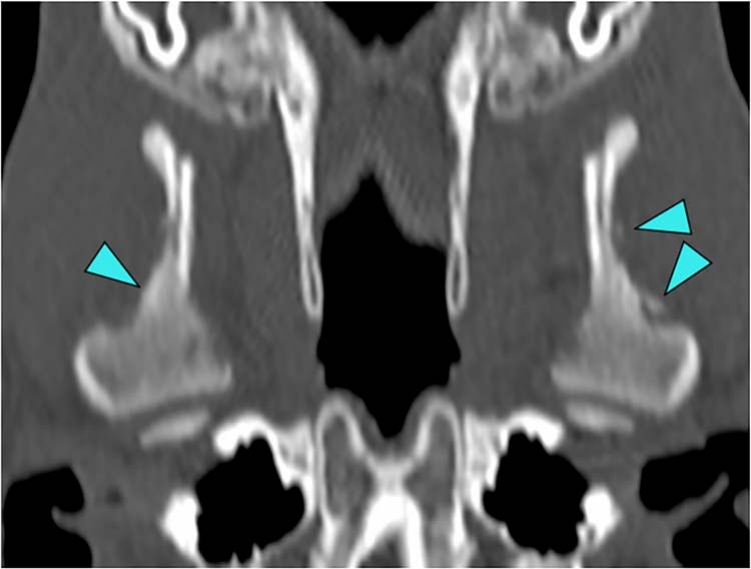
Skull computed tomography scan image in the dorsal plane. Arrows indicate irregular bone surface with partial cortical discontinuity of mandibular ramus.

The dog was treated empirically with amoxicillin/clavulanic acid IV (*Co-amoxiclav,* Bowmed Ibisqus, Wrexham, UK; 20 mg/kg q8h) because of concern about aspiration pneumonia, which was suspected based on the history of vomiting and clinical findings, including pyrexia, tachypnea, and coughing.

The dog also received IV fluids (*Aquapharm No. 1 0.9% Sodium-Chloride*, B.BRAUN, Melsungen, Germany; 4 mL/kg/h), maropitant (*Vetemex*, Virbac, Burgdorf, Germany; 1 mg/kg IV), and furosemide (*Dimazon*, MSD Animal-Health, Dublin, Ireland; 1 mg/kg q12h IV). On day 2, the venous iCa concentration was increased (2.31 mmol/L), with decreased serum sodium (135 mmol/L) and potassium (2.7 mmol/L; RI, 3.3-4.9) concentrations. The dog was discharged at the owner’s request, but was readmitted 2 days later because of persistent anorexia and inability to take PO medication. Sedation with butorphanol (*Torbugesic,* Zoetis, Girona, Spain; 0.2 mg/kg IV) allowed placement of a naso-esophageal feeding tube (*Nutrisafe2*, Vygon, Swindon, UK 75 cm 8 French) and a nasal oxygen catheter. Blood tests on readmission showed increased iCa (2.29 mmol/L), urea (36 mg/d,L; RI 7‑26) and glucose (7.4 mmol/L, RI 4.5‑6.6) and decreased sodium (136 mmol/L), potassium (2.4 mmol/l), and chloride (104 mmol/L; RI, 109‑121 mmol/L) concentrations. Intravenous fluids with potassium supplementation (*Potassium Chloride 15%,* MercuryPharma, London, UK; 10 mmol/250 mL), maropitant (*Vetemex*, Virbac, Burgdorf, Germany; 1 mg/kg IV), continued antibiotics, oxygen (100 mL/kg/min), and enteral feeding (Royal-Canin Recovery Liquid, 1/3 resting energy requirements) were administered. Mild regurgitation prompted metoclopramide treatment (*Emeprid*, Ceva, Libourne, France; 0.3 mg/kg IV q12h).

Within 24 h, the respiratory rate and temperature normalized, and voluntary eating began. Repeat laboratory testing showed normalized serum sodium (144 mmol/L), chloride (113 mmol/L), and potassium (3.3 mmol/L) concentrations, and increased iCa (1.94 mmol/L) and urea (28 mg/dL) concentrations; the CRP was 18.9 mg/L. Dexamethasone (*Dexadreson*, MSD Animal-Health, Milton Keynes, UK; 0.05 mg/kg IV) was added in an attempt to control the persistent hypercalcemia, after clinical signs attributed to suspected pneumonia had resolved. Intravenous fluids were discontinued because drinking had resumed. Oral potassium (*Kaminox*, VetPlus Ltd, Lytham, UK; 2 mL/4.5 kg PO q8h) and antibiotics were continued. Twenty-four hours after dexamethasone, the serum iCa concentration decreased to 1.62 mmol/L, serum sodium and chloride concentrations were mildly decreased (136 and 107 mmol/L, respectively), and the serum potassium concentration normalized. By day 7, clinical status and physical findings were normal. The dog was eating voluntarily and was discharged on PO antibiotics (*Synulox*, Zoetis, Surrey, UK; 12.5 mg/kg q12h) and prednisolone (*Prednicare 5 mg*, Ecuphar, Oostkamp, Belgium; 0.75 mg/kg q24h PO) to complete a 14-day course.

Ultrasonography of the neck 2 days after discharge, under sedation with butorphanol (*Torbugesic*, Zoetis; 0.2 mg/kg IV) and medetomidine (*Sedator*, Dechra, Bladel, The Netherlands; 3 μg/kg IV) identified the largest parathyroid gland located at the mid-ventral aspect of the left thyroid gland, measuring 0.3 cm in length and 0.18 cm in width. Dorsal and slightly cranial to this gland was a second smaller left parathyroid gland measuring 0.16 × 0.09 cm (Figure 4). The right parathyroid glands were normal-sized and measured 0.16 × 0.09 cm and 0.28 × 0.08 cm. Twenty days after presentation the dog was referred to a second hospital for parathyroidectomy.

**Figure 4:**
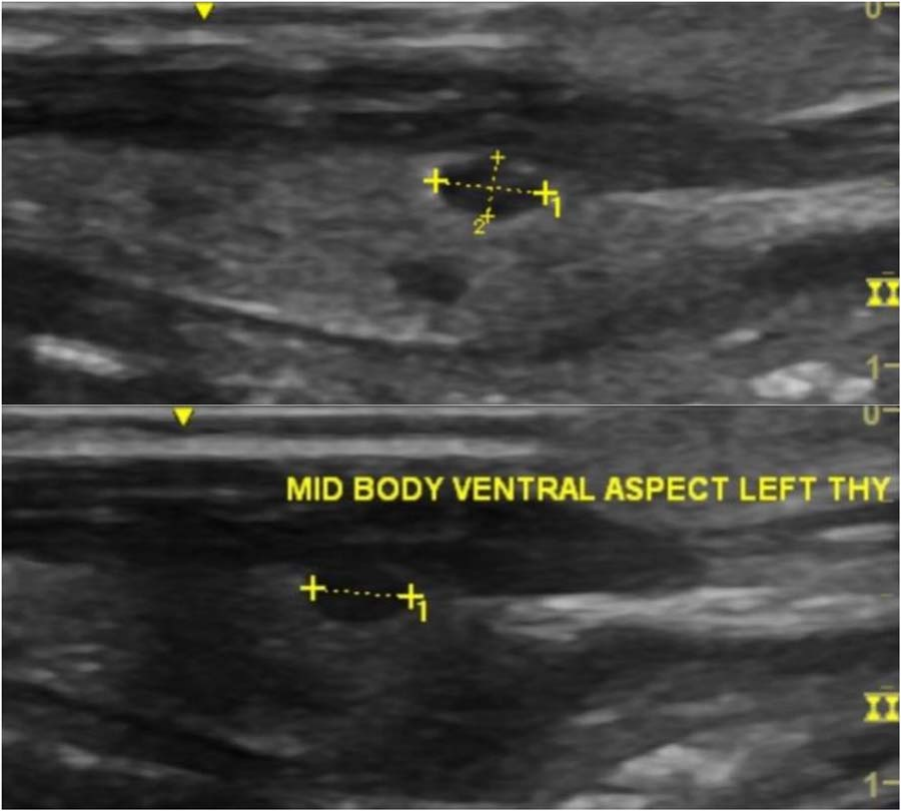
Sonographic images of the left thyroid gland.

A small nodule (approximately 3 × 2 mm) was identified on the mid-ventral aspect of the left thyroid gland, consistent with the internal parathyroid gland, and was excised together with a small portion of adjacent thyroid tissue. Two additional nodules were identified caudal to the left thyroid gland and were excised. Three samples were submitted for histopathologic evaluation, which identified 1 sample as thyroid gland tissue bordered by parathyroid gland tissue, consistent with the internal parathyroid gland. The other 2 samples consisted of adipose tissue containing portions of a lymph node.

The dog recovered uneventfully from general anesthesia and was monitored for signs of hypocalcemia. The serum iCa concentration decreased from 1.9 mmol/L (28 h preoperatively) to 1.47 and 1.55 mmol/L, 15 and 25 h after the surgery, respectively. At the time of the discharge, the serum iCa concentration was 1.6 mmol/L.

Histopathology of the resected parathyroid gland tissue showed changes compatible with chief cell hyperplasia (Figure 5). Serum iCa concentration measured 7 and 77 days after parathyroidectomy was within the RI, 1.37 and 1.4 mmol/L, respectively. The owner confirmed that the dog was back to normal, slowly re-gaining weight, normal appetite, and mobility. The dog was still sensitive on examination of its mouth, but the owner confirmed normal opening of the mouth and no dysphagia.

**Figure 5:**
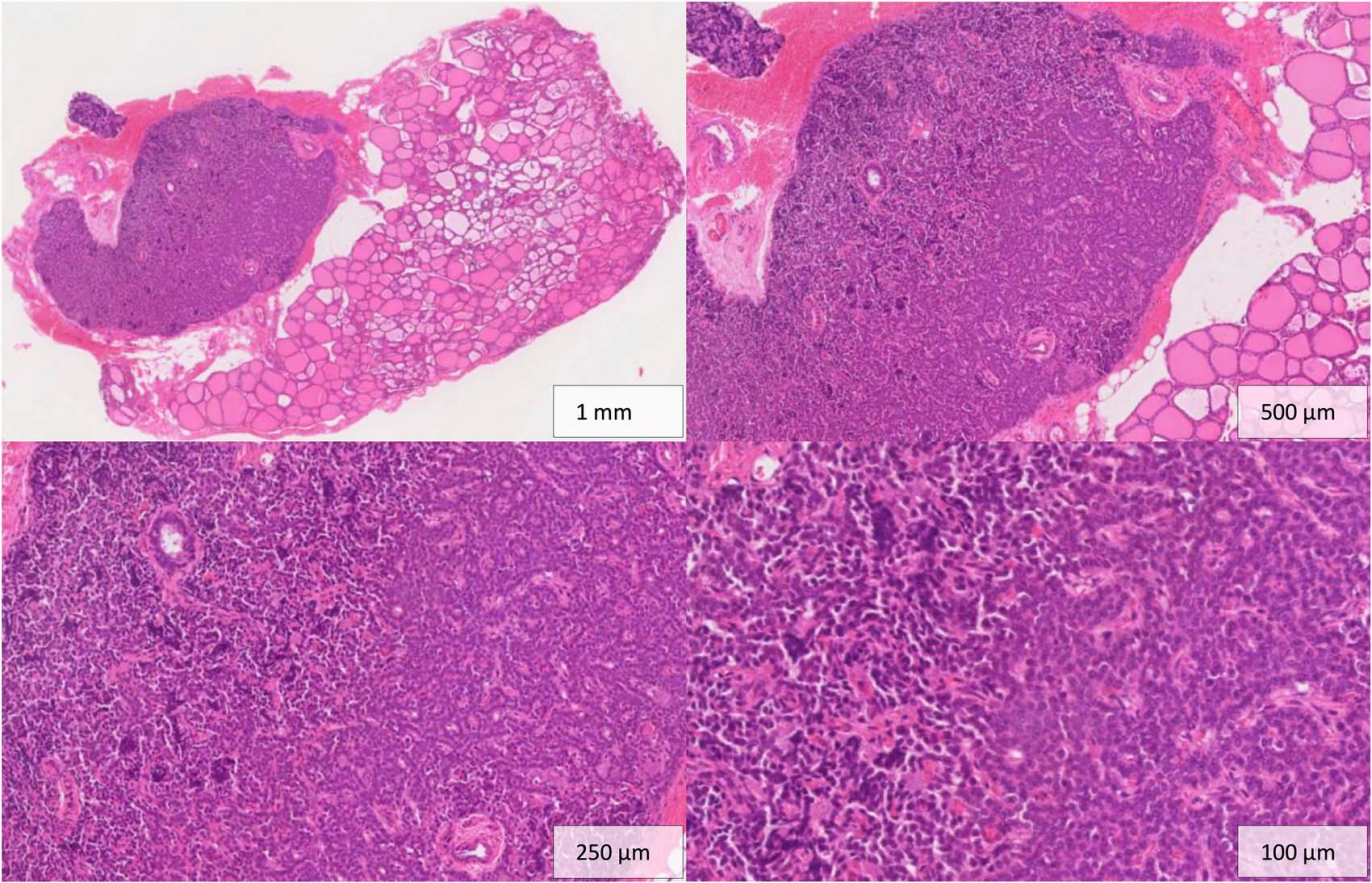
Thyroid and parathyroid gland. The parathyroid tissue exhibits changes consistent with chief cell hyperplasia. Magnification: 2.5×, 5×, 10×, and 20×, respectively.

## Discussion

Primary hyperparathyroidism is a rare endocrine condition in dogs, but is considered common in humans, affecting 0.84% of the population, particularly postmenopausal women.^16^ In contrast to adult-onset PHPT, juvenile PHPT is rare and reported to affect 2‑5 people out of 100 000 live births.^13^ Only 2 cases have been reported in veterinary medicine.^17^

Presenting clinical signs in our case were consistent with clinical signs reported in dogs with acquired and juvenile PHPT.^3^^,^^17^ Polyuria and polydipsia, as a result of impaired function of the distal tubule associated with hypercalcemia, seem to be the predominant clinical sign in juvenile PHPT, along with stunted growth and skeletal abnormalities.^17^ The puppy of our report had carpal valgus of both limbs, which could be breed and age specific.^18^ No further diagnostic tests were carried out to investigate this abnormality because the mobility of the puppy was normal.

Pyrexia, tachypnea, and coughing were most likely caused by pneumonia, which could have been related to aspiration secondary to vomiting before presentation. Canine infectious respiratory disease complex and pneumonia secondary to immune dysfunction cannot be ruled out.^19^ Computed tomography imaging did not identify typical lung patterns associated with aspiration pneumonia and the reported increased ground glass attenuation of the lung parenchyma also could be related to the patient’s age,^20^ but a lag in radiographic changes with regard to clinical resolution of the pneumonia must also be considered for the respiratory abnormalities. Pyrexia and CRP resolved with antibiotic treatment.

The main clinical abnormality in this dog was oral pain on examination and opening of the mouth. No dental or oral abnormalities were identified on examination. Therefore, we assumed the pain was related to bone defects along the articular surface of the mandibular condyles, mandibular neck and both rami, findings consistent with metabolic bone disease. Bone abnormalities and skeletal pain are the most common features of PHPT in children and are most likely a consequence of increased bone turnover.^13^^,^^15^ Radiographic changes reported in children are pseudo-fractures, subperiosteal resorption, metaphyseal irregularities, osteopenia and osteitis fibrosa cystica.^15^ Necropsy findings in the previously described affected German Shepherd puppies were consistent with fibrous osteodystrophy, with the most severe changes involving the ribs.^17^

Diagnosing hypercalcemia in our case was not straightforward although both serum total and iCa concentrations were increased based on the RI established for adult dogs.^21^ Young dogs can have serum calcium concentrations slightly above the RI established for adults, but the specific cut-off of what is considered abnormal as opposed to physiological, and whether applicable to both serum total and iCa concentration is to our knowledge not available. To diagnose hypercalcemia in the dog of our report, we combined published information.^21–23^ This approach also was applied to the PTH concentration. In another study, total calcium concentration was measured in all puppies from the same litter and hypercalcemia in the 2 affected puppies was diagnosed by comparing their calcium concentrations with those of 2 clinically normal puppies; serum iCa concentration was not measured in this study.^17^ Parathyroid hormone in this study was measured using the immunoassay and the PTH in both affected puppies was increased compared with results in clinically normal dogs.^17^

Localization of the hyperactive gland in children is a 2-step process consisting of ultrasonography of the neck followed by scintigraphy using technetium-99 m sestamibi.^13^^,^^24^ Ultrasonography of the neck remains the diagnostic imaging procedure of choice in dogs. Current optimal cut-off, allowing differentiation of hyperplastic and neoplastic parathyroid glands from normal glands, is <3 mm in diameter, but results overlap among groups of dogs with various abnormalities of the parathyroid glands.^5^ Given that parathyroid gland measurements are mostly available from adult dogs, scanning the puppy of our report we had to rely not only on the size of the gland, but also on the shape and echogenicity of the gland in comparison with the other parathyroid glands.

Presurgical serum calcium concentration was high, and an initial attempt to decrease serum calcium concentration using diuresis with saline combined with diuretics did not result in measurable improvement. The owner elected to take the dog home after 1 day, which may have influenced the outcome. Administration of a low dose of corticosteroids, introduced after resolution of the clinical signs associated with suspected pneumonia, helped resolve the persistent hypercalcemia. Presurgical treatment options in children include parenteral isotonic solutions, calciomimetic agents, bisphosphonates, calcium-poor diets, and corticosteroids.^15^

Another preoperative abnormality was a markedly increased urine protein:creatinine ratio. The cause of the proteinuria was not further investigated during admission or follow-up, representing a limitation of our report, because the persistence or resolution of proteinuria after recovery could not be determined. Proteinuria is rare in children and adults with PHPT and most likely is associated with renal damage caused by hypercalcemia.

Surgical removal remains the treatment of choice for PHPT in dogs, providing direct visual evaluation and palpation of all parathyroid glands and facilitating complete excision of hyperfunctional tissue while preserving the normal glands,^25^ and with reported success rates of 92%‑95%.^26^ In pediatric human patients, parathyroidectomy remains the preferred treatment.^13^^,^^27^ Minimally invasive approaches can be used when intraoperative PTH monitoring is available.^27^Abnormal tissue was submitted for histopathology and identified mild to moderate chief cell hyperplasia. This finding is in accordance with a previous report of 1 puppy with juvenile PHPT, but in contrast with juvenile PHPT in children and adults in whom a solitary adenoma is the most common histologic finding.^13^^,^^17^

The dog of our report recovered well after surgery, serum calcium concentration remained increased above the RI for several days postoperatively, but normalized 7 days postoperatively and remained normal a few months later. Oral discomfort was still present on day 7 and the dog still resented examination of the mouth 77 days postoperatively. Bone pain is well described in humans with PHPT and resolves a variable period of time after surgery. Bone discomfort caused by bone remodeling associated with increasing bone density after resection of the parathyroid adenoma also can reoccur after initial resolution. ^28^^,^^29^

Primary hyperparathyroidism should be considered in puppies with increased serum iCa concentration and concurrent bone pain or bone deformities. Surgical resection of the abnormal gland (or glands) leads to a complete resolution of the hypercalcemia, but bone discomfort and deformities might take a longer time to resolve. Further research on similar cases will be required to establish the skeletal manifestations of this disease in puppies and the necessary recovery time after surgery.

## Off-label antimicrobial declaration

Authors declare no off-label use of antimicrobials.

## Institutional animal care and use committee or other approval declaration

Authors declare no institutional animal care and use committee or other approval was needed.

## Abbreviations

PHPT: primary hyperparathyroidism
PTH: parathyroid hormone
PTHrP: parathyroid hormone-related protein
